# Peach ( *Prunus persica* ) TAC1 protein interaction with a LIGHT HARVESTING CHLOROPHYLL A/B BINDING (LHCB) homolog and transcriptomic analyses reveal connections to photosynthesis

**DOI:** 10.17912/micropub.biology.001371

**Published:** 2025-02-19

**Authors:** Jessica M. Waite, Erik Burchard, Chris Dardick, Courtney A. Hollender

**Affiliations:** 1 USDA ARS Tree Fruit Research Laboratory; 2 USDA ARS Appalachian Fruit Research Station; 3 Michigan State University Department of Horticulture

## Abstract

Plants receive and interpret external light, gravity, and temperature cues to both set and change the angles of their lateral organs for optimal growth and development. In recent years, the roles of the IGT/LAZY protein family in integrating light and gravity cues have become increasingly apparent. Here we investigated protein-protein interactions for peach ( *Prunus persica* ) TAC1 (PpeTAC1). *TAC1* is a light-regulated IGT/LAZY family member involved in maintaining outward growth of lateral branches in numerous plant species. Using a yeast-two-hybrid screen with a peach library, we identified three candidate protein interactors, including a LIGHT HARVESTING CHLOROPHYLL A/B BINDING (LHCB) family protein. We found that neither *TAC1* silencing nor *PpeTAC1* overexpression in plum ( *P. domestica* ) altered chlorophyll content, despite a recent finding that *LAZY1* -silenced plum trees have chlorotic leaves due to reduced chlorophyll. However, we identified multiple differentially expressed chloroplast-, photosynthesis-, and light-related genes between *tac1* mutant and standard peaches. Collectively, we identified connections between *PpeTAC1* and chloroplasts, photosynthesis-related machinery, and light. This data supports a role for the TAC1 protein as an integrator of light perception into mechanisms controlling lateral organ orientation in concert with or in parallel to the LAZY/DRO gravitropic-response pathway.

**Figure 1. PpeTAC1 connections with photosynthesis- and plastid-related genes, as shown through yeast-two-hybrid interactions, co-expression, and comparative transcriptomics:**
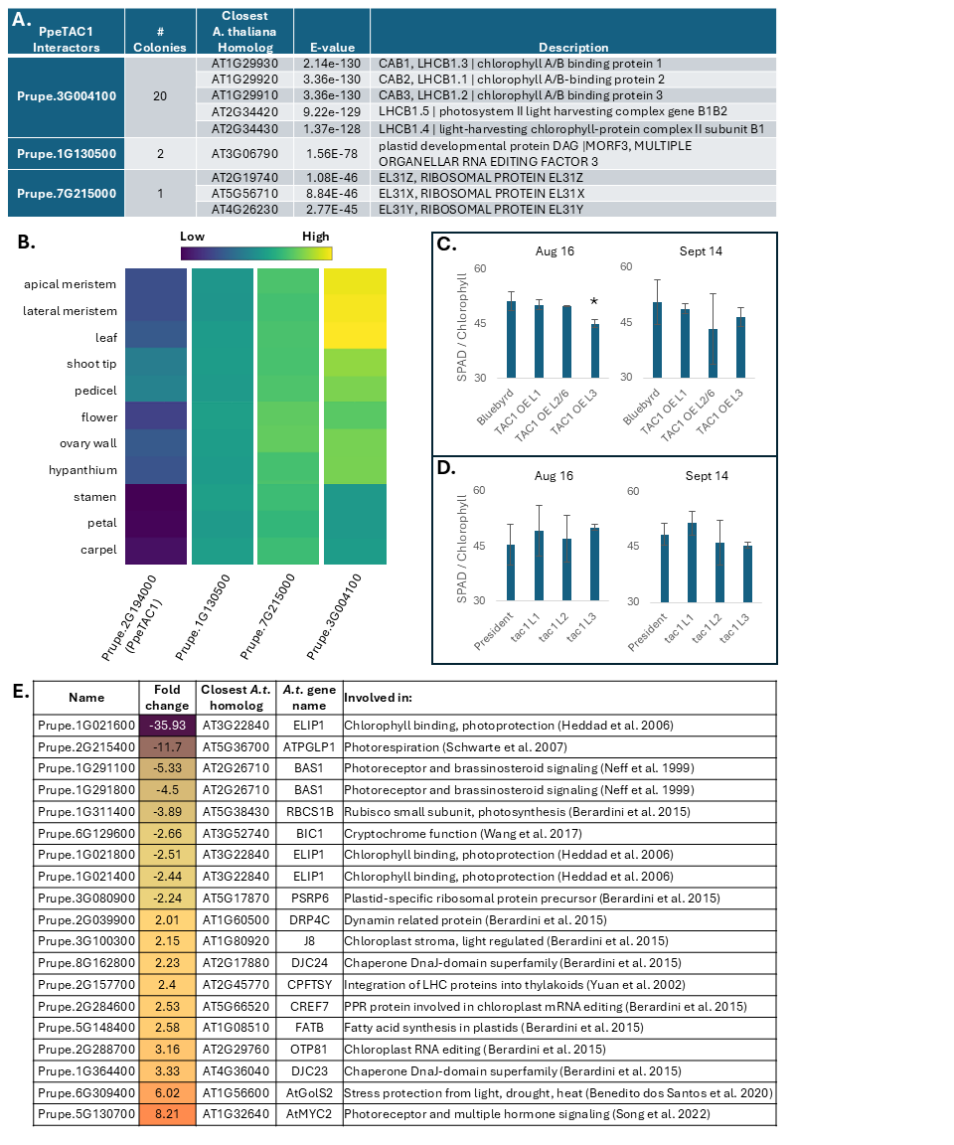
**A** . Candidate protein interactor results from a yeast-two-hybrid screen using peach TAC1 (PpeTAC1) as bait. Peach gene IDs from the *Prunus persica* v2.0 assembly (Verde et al. 2017) are reported along with the number of yeast colonies from which each interactor was identified. Closest *Arabidopsis thaliana* homologs for each gene, as well as E-values and brief protein descriptions are also shown. **B.** Gene expression of *PpeTAC1* and the three identified interactors across multiple types of aerial tissues. Heatmap visually demonstrates that all genes are co-expressed at similar (medium expression) levels in shoot tips and pedicel tissues. Co-expression also occurs for shoot meristems and some floral tissues, but with a greater range of expression. Scale is based on normalized, log transformed transcript read counts. **C and D** . Chlorophyll measurements in plums ( *Prunus domestica* ) with *PpeTAC1* overexpressed (C) or *TAC1* silenced (D) exhibited no major changes in chlorophyll content compared to controls. Error bars represent standard deviation for three trees of each line. Asterisk (*) indicates significance (p ≤ 0.05) according to a Student’s t-test. **E.** Chloroplast-, photosynthesis-, and light-related differentially expressed genes from a transcriptomic comparison between *Ppetac1* mutant and standard peaches. *P. persica* genome v2.0 gene IDs and fold change in *Ppetac1* relative to standard trees are reported, along with closest Arabidopsis homologs and a short description of known processes that these genes are involved in.

## Description

Members of the *IGT/LAZY* gene family, including *TILLER ANGLE CONTROL 1* ( *TAC1* ), *LAZY* , and *DEEPER ROOTING* ( *DRO* ) genes, have emerged as key players in integrating light and gravity cues to set the orientation of plant organ growth (Kawamoto et al. 2022, Waite et al. 2021, Waite et al. 2018) . The *IGT/LAZY* genes in peach ( *Prunus persica* ) were identified when a transposon insertion in *PpeTAC1* was determined to cause a columnar peach growth habit resulting from a narrow branch angle phenotype (Dardick et al 2013) . Silencing of *TAC1* via RNAi in plum ( *Prunus domestica* ) phenocopied the peach columnar growth habit while overexpression resulted in wider branch growth angles (Hollender et al 2018) . The upright branch angle phenotype of *tac1* loss-of-function or silenced plants across species supports its role in promoting wide branch angles, which is opposite to *lazy1* mutants with wide to weeping branch growth (Xu et al. 2017, Hollender et al. 2018, Hollender et al. 2020, Li et al 2022a, Kawamoto and Morita 2022, Kohler et al. 2024). While *TAC1* plays a key role in setting the angle of lateral branch growth, or gravitropic set point angle, it was not required for gravitropic reorientation responses in *Arabidopsis thaliana* (arabidopsis) (Hollender et al. 2020) . In contrast, research over the past few decades has determined that LAZY and DRO clade proteins are central to organ reorientation in response to changes in gravity (Kawamoto and Morita 2022, Yoshihara et al. 2020) . Mechanistically, DRO proteins have been shown to direct the asymmetric auxin transport associated with gravitropic responses in arabidopsis roots. DRO1, 2, and 3, are also referred to as LZY3, 4, and 2 in Nishimura et al. 2023, and LAZY4, 3, and 2 in Yoshihara and Spalding 2017, respectively. Gravistimulation leads to their phosphorylation and binding to amyloplasts during the sedimentation process, followed by the recruitment of RCC1-like domain (RLD) proteins to the plasma membrane, which regulate PIN-dependent auxin transport (Furutani et al. 2020, Nishimura et al. 2023, Chen et al. 2023, Kulich et al 2023) . Specific amino acid alterations to LAZY1 domain II, or the IGT domain, was also shown to reverse gravitropism in arabidopsis branches (Yoshihara et al. 2020) . Significantly less is known about how TAC1 functions in branch angle and orientation control. TAC1 proteins contain four out of five conserved domains found in LAZY proteins (domains I-IV). However, they lack the C-terminal EAR-like CCL domain V, which is important for LAZY and DRO protein interactions with RLD proteins (Waite and Dardick 2021) .

In addition to their roles in gravitropism, multiple lines of evidence connect IGT/LAZY family members with light signaling. *AtTAC1* expression is light-dependent and treatments with photosynthesis inhibitors drastically reduce its expression and lead to a narrow branch angle phenotype (Waite et al. 2018) . Additionally, in rice, *OsTAC1* expression responds to photoperiod, which contributes to altered plant architecture (Wang et al. 2023) . LC-MS/MS screens for protein-protein interactors of IGT family members DRO1 and DRO3 revealed multiple light- and photosynthesis-related candidates: LIGHT HARVESTING CHLOROPHYLL A/B BINDING protein, LHCB4.1, and two proteins associated with Photosystem I (PSI) (Furutani et al. 2020) . *LAZY* and *DRO* genes are also required for light-induced changes to lateral organ angles, and *AtDRO1* expression is light regulated via repression by PHYTOCHROME INTERACTING FACTORS in the hypocotyl and promotion by ELONGATED HYPOCOTYL 5 (HY5) in the root (Waite et al. 2024a, Yang et al. 2020) .

To better understand *TAC1* function in trees, we developed a yeast-two-hybrid (Y2H) library of peach proteins to identify putative protein interactors for PpeTAC1 protein (Prupe.2G194000). The PpeTAC1 bait vector was transformed into yeast, mated with the Y2H prey library, and plated on appropriate selective media. Positive colonies were selected, re-plated on higher stringency media, and plasmids from surviving colonies were sequenced to determine the identity of candidate interacting proteins. We found 23 putative interactors with the PpeTAC1 bait. Sequencing results identified three distinct genes ( Fig. 1A ). Prupe.3G004100, whose closest arabidopsis homologs belong to the LIGHT HARVESTING CHLOROPHYLL A/B BINDING 1 (LHCB1) protein family, accounted for 20 of the 23 interactions ( Fig. 1A ). LHCB1 proteins are important for photosynthesis, through regulation of photosystem efficiency and photoprotection (Dall’Osto et al. 2015). They are synthesized in the cytosol before being post-translationally imported to the chloroplast thylakoid membrane, where they form parts of a modular light harvesting complex (LHC) antenna system that surrounds photosystem II (PSII) reaction centers (Dall’Osto et al. 2015, Pietrykowska et al. 2014). Two additional interactors were identified, accounting for 3 of the 23 interactions, Prupe.1G130500 and Prupe.7G215000 ( Fig. 1A ). Prupe.1G130500 is most closely related to MULTIPLE ORGANELLAR RNA EDITING FACTOR 3 (MORF3), and Prupe.7G215000 to ribosomal proteins in the L31e family. MORF proteins are involved in RNA editing in mitochondria and plastids, changing cytidines to uridines, largely in mRNA coding regions (Takenaka et al. 2012) . MORF3 is required for this process in mitochondria, whereas MORF2 and MORF9 carry this out in chloroplasts. Arabidopsis homologs of Prupe.7G215000, EL31Y, X, and Z, belong to the L31e family of cytoplasmic ribosomal proteins, and form part of the large 60S ribosomal subunit (Scarpin et al. 2022) . To address whether interactors are expressed in a similar spatiotemporal context, we analyzed tissue-specific expression across published datasets (see methods). Transcripts from these datasets were collectively normalized such that transcriptomic comparisons could be made. We found that *PpeTAC1* was expressed at similarly high levels to all interactors in shoot tips and flower pedicels ( Fig. 1B ). Co-expression, but to a lesser degree occurred in apical and lateral meristems, and some floral tissues ( Fig. 1B ).

To further investigate the connection between *TAC1* and chloroplasts, we next tested whether chlorophyll levels were altered in plum trees with *TAC1* silencing or ectopic expression of *PpeTAC1* . A recent long-term study on *LAZY1-silenced* plums showed seasonal reductions in chlorophyll content (Kohler et al. 2024) . Chlorophyll content was assessed using a SPAD chlorophyll meter. We found no significant differences between the *TAC1* genotypes and standard trees at either timepoint ( Fig. 1C and 1D), suggesting that association between PpeTAC1 protein and the LHCB1 homolog Prupe.3G004100 may not be important for chlorophyll accumulation.

Additionally, given associations between *TAC1* , photosynthesis, and light, we mined published transcriptome data from standard and *tac1* mutant peach trees (Hollender et al. 2018) to reassess differential expression of photosynthesis- and plastid-related genes. 28 genes described as chloroplast- or light-related were identified, 19 of which had a positive or negative fold change of two or greater ( Fig. 1E ). These included downregulation of three homologs of *EARLY LIGHT INDUCIBLE PROTEIN 1* ( *ELIP1* ): *Prupe.1G021600* (-35X), *Prupe.1G021800* (-2.5X), and *Prupe.1G021400* (-2.4x). ELIP1 proteins co-isolate with arabidopsis LCHB proteins in the PSII antenna under high light, and *ELIP1* expression increases as photosystem damage increases (Heddad et al. 2006) . Interestingly, Prupe.1G021800 was identified as a candidate interactor of PpeDRO1 protein (Waite et al. 2024b) . Other notable downregulated differentially expressed genes (DEGs) include two homologs of *PHYB ACTIVATION TAGGED SUPPRESSOR 1 (BAS1)* , a cytochrome p450 family member that mediates signaling between photoreceptors and brassinosteroid (BR) signal transduction (Neff et al. 1999) ; a homolog of *BLUE-LIGHT INHIBITOR OF CRYPTOCHROMES 1 (BIC1)* , a cryptochrome inhibitor involved in blocking cryptochrome dimerization in response to blue light (Wang et al. 2017) ; a Rubisco small subunit gene; and a homolog of *PGLP1* , a 2-phosphoglycolate phosphatase required for photoprotection through conversion of phosphoglycolate, a toxin produced by photorespiration (Schwarte et al. 2007) . Notable upregulated DEGs include: a homolog of *AtMYC2* , which is involved in signal integration between photoreceptors and multiple hormone pathways, including regulation of light-inducible genes (Song et al. 2022, Yadav et al. 2005) ; a homolog of *GALACTINOL SYNTHASE 2 (AtGolS2)* , a gene associated with increased tolerance to light, heat, and drought stresses (Benedito dos Santos et al. 2020); and a homolog of *CPFTSY* , which is involved in integrating proteins into Light Harvesting Complexes (Yuan et al. 2002) . We also addressed whether any of the candidate interactors of PpeTAC1 were differentially expressed in this dataset, and the genes for these interactors did not appear in the DEG list. The altered expression in these genes might suggest that TAC1 acts upstream of chloroplast and photosynthesis genes. However, the upright branch growth phenotype of these plants could play a more indirect role in these changes, which we are unable to distinguish at this time.

Together, the PpeTAC1 Y2H interaction with a LHCB protein and the altered expression of multiple chloroplast- and photosynthesis-related proteins in a *PpeTAC1* -silenced background further supports a role for TAC1 in integrating light and gravity cues for branch orientation control. Paired with the finding that *TAC1* expression in arabidopsis is highly regulated by photosynthetic pathways, *TAC1* may be involved in feedback loops regarding light-regulation of branch angle, however further experimentation is required. Arabidopsis DRO1 and DRO3 putative interactions with LCHB4 and PSI proteins suggest a common connection between IGT/LAZY proteins and photosynthetic pathways (Furutani et al. 2020) .

## Methods

**Y2H library construction**

Peach RNA was extracted from mature trees of the cultivar “Encore”, using Trizol reagent (ThermoFisher Scientific). An Oligotex kit (Qiagen) was used to enrich mRNA in a 2mg sample of total RNA, finally yielding 20ug of mRNA. To make the Y2H cDNA library, 8ug of mRNA was input into a HybriZAP®-2.1 two-hybrid cDNA gigapack cloning kit and HybriZAP®-2.1 two-hybrid cDNA synthesis kit (Agilent Technologies) including modifications: (1) Invitrogen Superscript III Reverse Transcriptase (ThermoFisher Scientific) was used as the enzyme; (2) cDNA purification was performed using Amersham GFX columns; (3) cDNA size selection was performed to remove cDNAs <600bp. 150ng of size selected cDNA was ligated and 1uL of cDNA was used for phage packing five times to build the library. The library was then amplified, stored at -80C, and phagemid was excised. To screen the library, amplified phagemid was purified using the Qiagen Maxi prep kit (Qiagen) and co-transformed with linearized pGADT7 vector (the Y2H prey vector with activation domain) into the yeast strain Y187 via the Yeast transformation kit, Frozen-EZ yeast transformation II (Zymo Research).

**Yeast-two-hybrid Bait construct cloning**

Y2H Binding Domain (BD/bait) vectors were constructed using the pXDGATcy86 backbone (Ding et al. 2007) . The full length peach *TAC1* CDS sequence was amplified by RT_PCR from peach RNA using the following primers: PpeTAC1-CDS-EcoRI-F (5’ GAA TTC ATG AAG ATC TTC AAC TGG GTT CAT A) and PpeTAC1-CDS-BamHI-R (GGA TCC TCA GTG CAC ACA AGG GG). The amplicon was sub-cloned into Invitrogen pENTR/D-TOPO (ThermoFisher Scientific, Waltham MA), sequenced, and then cloned into the pXDGATcy86 backbone vector using LR clonase, resulting in PpeTAC1-BD. The PpeTAC1-BD vector was then transformed into the yeast strain AH109.

**Yeast-two-hybrid mating and selection**

Yeast containing the PpeTAC1-BD vector (AH109) and prey library vectors (Y187) were mated and plated according to the Clontech Yeast Protocols Handbook PT3024-1 (Clontech, now Takara Bio USA) and as previously described in Waite et al 2024b. Briefly, AH109 bait cultures were grown overnight, centrifuged, and resuspended in YPD media to a concentration of ~2x10e8 cells. Library/prey aliquots containing ~1x10e8 cells were added to the bait cultures, then incubated at 30C for 3-4 hours with shaking. Liquid was removed, leaving yeast on a filter membrane, which was placed yeast-side-up on YPD media plates (pH 4.5), then incubated overnight at 30 ^o^ C. Membranes were washed and yeast was collected and plated on selection plates (SD -His, -Leu, -Trp +3AT), reserving some yeast for serial dilutions and calculation of library size. Growing colonies were picked and re-streaked on SD -Trp and SD -His, -Leu, -Trp plates. DNA was then extracted from these yeast strains and sequenced. Sequences were used for a BLAST search on the Genome Database for Rosaceae (https://www.rosaceae.org) against the peach genome (v2.0) to determine interactor identity (Verde et al. 2017, Jung et al. 2019) .

**Plant materials**

Peach trees used for expression analyses were derived from a self-pollinated tree heterozygous for the pillar trait (KV991636) in 2002. For *TAC1* RNAi silenced plums and *PpeTAC1* OE plums, trees were generated as described in Hollender et al. 2018. Briefly, RNAi silencing vectors were developed by amplifying a 380bp fragment from peach cDNA, and cloning it into the pHellsgate 8 vector (Commonwealth Scientific and Industrial Research Organisation (CSIRO), Australia), using gateway cloning technology. OE vectors were developed by cloning the *PpeTAC1* coding sequence into a modified pBIN-ARS vector (called pBIN-AFRS) containing the 35S Cauliflower Mosaic Virus promoter, using restriction cloning methods. Vectors were transformed into plum hypocotyl slices using Agrobacterium strain GV3101, as described in Petri et al. 2012. Seedlings derived from open-pollinated “Bluebyrd” and “President” trees were used as a source of hypocotyl material and as standard controls.

**Chlorophyll measurements**

Chlorophyll was measured at two time points during the summer of 2018, August 16 ^th^ and September 14 ^th^ . Three independent lines from plum transformation were used for each of *PpeTAC1* OE and *TAC1* RNAi silences plums. Measurements were taken from 3 different locations in the canopy, using 3 trees from each line with a SPAD 502 Chlorophyll Meter (Konica Minolta, Inc., Tokyo, Japan). Measurements from each tree were averaged, then the 3 trees from each line were averaged and reported, with statistical tests.

**Tissue-specific gene expression**

Generally, tissue was collected in liquid nitrogen from greenhouse or field grown trees. Tissues were dissected by hand and ground to powder in liquid nitrogen, or lyophilized and ground using a homogenizer. RNA was extracted using either a Norgen Plant/Fungi Total RNA Purification Kit (Norgen Biotek, Thorold, ON, Canada) or an Omega SQ Total RNA kit with 2% polyvinylpyrrolidone added to the red cell lysis buffer (Omega Bio-tek, Norcross, GA). RNA sequenced using Illumina Hi-Seq. Sequence analysis and heatmap generation methods were described previously (Waite et al. 2024b) . Briefly, multiple RNA sequence datasets were analyzed to cover multiple tissue types. Differential gene expression from these datasets has been published previously: data from shoot tips were included in (Hollender et al. 2018) ; hypanthium and ovary wall in (Li et al. 2022) ; flower, petal, carpel, and stamen in (Zhu et al. 2020) ; lateral meristem, apical meristem, and pedicel in (Waite et al. 2024b) . Raw RNA reads were re-mapped to the Prunus persica v2.0 genome (Verde et al. 2017) using STAR v2.7.10a transcript alignment software with default parameters (Dobin et al. 2013) . The featureCounts v2.0.3 tool from the Subread software package was used for transcript counts (Liao et al. 2014) . Counts were normalized using TMM (Trimmed Mean of M-values) to calculate effective library size using the R (v4.1.0, https://www.r-project.org) package edgeR’s (v3.34.1) calcNormFactors function (Robinson et al., 2010) . Mean log counts per million (CPM) was calculated for each tissue type for each gene of interest, and R package heatmaply, v1.4.2 was used to generate the heatmap, using mean values as input.

**Comparative transcriptome analysis between *tac1* mutant and standard peaches**

Transcriptome experimental procedures, dataset, and DEG analysis have been published and described previously (Hollender 2018) . Here, the descriptions of all DEGs found in the dataset between *tac1* and standard peach trees were subjected to searches for “chloroplast”, “light”, “photosynthesis”, and “plastid”. These were then filtered for fold change above 2 and below -2. Processes that these gene are involved were identified from the literature. In cases where there was no literature, descriptions found from The Arabidopsis Information Resource (TAIR) were used (Berardini et al. 2015) .
